# Influence of Framework Material and Abutment Configuration on Fatigue Performance in Dental Implant Systems: A Finite Element Analysis

**DOI:** 10.3390/medicina60091463

**Published:** 2024-09-06

**Authors:** Meryem Erdoğdu, Mehmet Gökberkkaan Demirel, Reza Mohammadi, Neslihan Güntekin, Masoud Ghanbarzadeh Chaleshtori

**Affiliations:** 1 Department of Prosthodontics, Faculty of Dentistry, Necmettin Erbakan University, 42090 Konya, Türkiye; kaandemirel@erbakan.edu.tr (M.G.D.); neslihanvarolnv94@gmail.com (N.G.); 2 Faculty of Dentistry, Necmettin Erbakan University, 42090 Konya, Türkiye; reza.mohammadi@ogr.erbakan.edu.tr; 3 Department of Mechanical Engineering, Buali University of Hamedan, Hamedan 65178-38695, Iran; masoud.gh71@yahoo.com

**Keywords:** fatigue performance, finite element analysis, implant-supported prostheses, von Mises stress

## Abstract

*Background and Objectives*: This study uses finite element analysis to evaluate the impact of abutment angulation, types, and framework materials on the stress distribution and fatigue performance of dental implant systems. *Materials and Methods*: Three-dimensional models of maxillary three-unit fixed implant-supported prostheses were analyzed. Abutments with different angles and types were used. Two different framework materials were used. Conducted on implants, a force of 150 N was applied obliquely, directed from the palatal to the buccal aspect, at a specific angle of 30 degrees. The distribution of stress and fatigue performance were then assessed, considering the types of restoration frameworks used and the angles of the abutments in three distinct locations. The simulation aspect of the research was carried out utilizing Abaqus Software (ABAQUS 2020, Dassault Systems Simulation Corp., Johnston, RT, USA). *Results*: In all models, fatigue strengths in the premolar region were higher than in the molar region. Maximum stress levels were seen in models with angled implants. In almost all models with the zirconia framework, fatigue performance was slightly lower. *Conclusions*: According to the findings of this study, it was concluded that the use of metal-framework multi-unit restorations with minimum angulation has significant positive effects on the biomechanics and long-term success of implant treatments.

## 1. Introduction

Dental implant abutments play a crucial role in the success and longevity of implant-supported prostheses. The type and angle of abutments have been shown to significantly impact stress distribution, mechanical stability, and peri-implant tissue health [1,2,3]. The angle of the dental implants is a critical factor that influences their placement accuracy, biomechanical behavior, and long-term success [1,4,5]. Angled implants offer both advantages and disadvantages in various medical and dental applications. One significant advantage of angled implants is their ability to compensate for non-ideal implant positions, allowing for better alignment with the natural anatomy and improving functional prosthesis outcomes [6]. By tilting the distal implant, a more posterior position can be achieved, enhancing implant anchorage by utilizing the cortical bone of the sinus and nasal fossa [7]. This positioning allows for longer implants in available bone, increasing the bone–implant contact area and primary anchorage [8]. Biomechanically, tilted implants can improve stability by increasing contact with cortical bone, leading to enhanced stability [9]. Additionally, the use of tilted implants can increase the anterior–posterior spread, contributing to a favorable pattern of bone resorption and stress distribution, especially when splinted with a rigid superstructure [10]. Research has shown that angled implants, when used in conjunction with a short cantilever, can decrease stress on peri-implant cortical bone, further enhancing the long-term success of the implant treatment [10]. Furthermore, the use of tilted implants can reduce costs by avoiding the need for angulated abutments, benefiting both clinicians and patients economically. However, angled implants also come with drawbacks. In terms of prosthetic considerations, tilted implants can increase stress on bone tissue and prosthetic screws, which should be carefully managed to ensure long-term success (Almeida et al., 2022). Another major disadvantage is the potential transfer of unfavorable forces to the implant or bone, which can compromise treatment prognosis by causing stress concentrations and damage to the peri-implant bone [11]. Moreover, concern is the increased cost, manufacturing complexity, and maintenance requirements associated with using angled abutments or bars to compensate for implant angulation [12]. Tao et al. [5] have highlighted that deviations in dental implant angles affect the success of the procedure and showed that implants with smaller deviations lead to better outcomes. Similarly, Mosharraf et al. [1] highlighted the impact of abutment angles on stress distribution in implant-supported prostheses, emphasizing the importance of considering angles in the design and placement of dental implants. Furthermore, Kitagawa et al. [13] investigated the influence of taper angle on the dynamic behavior of taper joint dental implants, emphasizing the importance of considering the angle in the design and analysis of implant systems. Other studies have utilized finite element analysis to investigate the effects of angle implantation cases on implant success, highlighting the biomechanical implications of implant angles [14,15]. These findings underscore the significance of dental implant angles in ensuring optimal biomechanical performance and long-term success. Additionally, a higher probability of failure and increased peri-implant marginal bone loss in dental implants connected to angled abutments compared to straight abutments have been observed, indicating the potential risks associated with certain implant angles [16]. The precise compensation of dental implant angles with the appropriate abutment type is crucial for the success of implant-supported prostheses. The angulation of implants and the type of abutment used can significantly impact the retention and stress distribution in the prosthesis. In this context, Frantz et al. [17] conducted an in vitro study to test the retention of overdenture attachment systems with different implant angulations and abutment types, highlighting the importance of considering the angle of the implants and the type of abutment for optimal retention. Additionally, Mosharraf et al. [1] performed a finite element analysis to evaluate the effect of abutment angulation on stress distribution in implant-supported prostheses, emphasizing the significance of considering different abutment angles for stress management.

Additionally, the choice of abutment material, such as ceramic or metallic, can influence peri-implant clinical parameters and tissue adaptation [18,19]. The choice of abutment material plays a crucial role in the overall outcome. Accordingly, a study investigated the effect of zirconia and alumina implant abutments on the light reflection of supporting soft tissues, indicating the importance of considering the material of the abutment for aesthetic and biological reasons [20]. Moreover, another study conducted a three-dimensional finite element analysis to assess the influence of prosthesis type and material on stress distribution in bone around implants; they emphasize the need to consider the material properties of the abutment for optimal stress distribution [21].

When deciding between a cemented or screwed abutment for an implant-supported prosthesis, several factors need to be considered. Cemented abutments offer advantages such as providing a more passive fit due to compensating for any distortion during framework casting [22]. However, it is crucial to note that the screw access channel in the abutment is commonly occluded prior to cementation, which can lead to challenges in case of future maintenance or repairs [23]. On the other hand, screwed abutments can help prevent issues like abutment screw loosening, which is a common problem with implant-supported prostheses [24,25]. Additionally, the choice of cement plays a significant role in the retention of restorations, with more retentive cements potentially causing damage to the implant fixture, abutment, or prosthesis if aggressive techniques are used for removal [26]. Moreover, the design of the abutment, including factors like height, taper, and margin level, can influence the retention and amount of residual cement in cement-retained restorations [27,28]. Studies have shown that the margin level of the abutment can impact the occurrence of residual cement, with subgingival margins leading to higher rates of cement residues [27]. Furthermore, the use of resin cements and different abutment types can affect the aesthetics of implant-supported restorations [29]. When considering abutment type for existing angulation in dental implant restorations, it is essential to weigh the advantages and disadvantages of screwed versus cemented abutments. Screwed abutments offer retrievability and ease of maintenance but they may be associated with higher rates of screw loosening, especially with increased abutment angulation [30,31]. On the other hand, cemented abutments provide better resistance to fluid and bacterial permeability, potentially leading to improved long-term outcomes [32]. However, it is crucial to note that the choice between screw-retained and cement-retained restorations should also consider factors such as aesthetics and occlusal integrity [33]. Studies have shown that abutment angulation plays a significant role in the stability and performance of implant restorations. Higher angulations have been linked to increased screw loosening and decreased mechanical strength, potentially leading to higher failure rates [31]. Additionally, the type of implant–abutment connection and screw length can influence preload retention and overall stability [34]. Angulated screw channel (ASC) abutments have been developed to allow for screw-retained restorations even in cases of non-ideal implant positioning, as long as the angulation correction needed is within a certain range [35]. As a result of these studies, it can be concluded that screwed abutments, especially those with angulation, offer benefits in terms of retention, stability, and adaptability to challenging anatomical situations. While some studies highlight potential issues like increased marginal bone loss with angulated abutments, the overall advantages of using screwed abutments in cases with angulation seem to outweigh the drawbacks. The effects of implant restoration materials on stress distribution have been extensively studied through various research articles. The choice of restoration material compositions, such as coping and veneering porcelain, has been shown to significantly affect stress distribution in implant-supported prostheses [1]. Additionally, the moduli of elasticity of implant restorative materials have been found to have similar effects on stress distribution through dental implants and their surrounding bone [36]. Furthermore, the use of composite resin materials as abutment or restoration materials has been suggested to resolve the dilemma of abutment rigidity under mechanical stress, indicating their potential to manage stress distribution in implant-supported restorations [37,38]. The influence of various occlusal materials on stress transfer to implant-supported prostheses and supporting bone has also been investigated. Studies have reported that the choice of occlusal surface material, such as acrylic-based materials, metal alloys, and ceramics, can impact stress distribution [39]. Moreover, the use of indirect composite materials with elastic properties as veneering materials for implant-supported prostheses has been suggested to improve the shock-absorbing capacity and reduce impact force and stress distribution on implants [40]. Finite element analysis (FEA) has been widely employed to simulate the stress distribution in dental implants and alveolar bone, providing valuable insights into the mechanical behavior of different restorative materials [41]. Additionally, the effect of prosthetic materials on crestal bone loss has been highlighted, with prosthetic materials found to have a greater effect on crestal bone loss than implant systems [42]. Implant restoration material compositions, specifically PFBM and PFBZ, have a significant impact on stress distribution within the prosthetic structures. Studies have shown that the choice of materials influences the stress distribution in implant-supported prostheses. For instance, Sertgöz Sevimay et al. [43] found that the combination of cobalt–chromium for the framework and porcelain for the occlusal surface resulted in optimal stress distribution in implant-supported fixed prostheses. Additionally, Bakitian et al. [44] reported that layered zirconia fixed dental prostheses (FDPs) are associated with more chipping of the veneering porcelain compared to PFBM FDPs, indicating a difference in stress distribution between these materials. Moreover, the literature suggests that the composition of the restoration materials impacts the fracture rates of the prostheses. Agustín-Panadero et al. [45] highlighted that PFBZ restorations have a fracture rate ranging between 6% and 15% over a 3- to 5-year period, while ceramo-metallic restorations exhibit fracture rates between 4% and 10% over 10 years. This indicates that the choice of materials, such as PFBZ restorations, can influence the longevity and success of the prostheses. In conclusion, the selection of implant restoration material compositions, whether PFBM or PFBZ, plays a crucial role in determining stress distribution within prosthetic structures. Understanding the impact of these material compositions on stress distribution is essential for designing durable and long-lasting implant-supported prostheses.

In conclusion, when dealing with existing angulation in dental implant cases, the choice between screwed and cemented abutments and also the choice between PFBM or PFBZ should be made based on a comprehensive assessment of factors such as maintenance ease, bacterial permeability, mechanical stability, and aesthetic considerations. It is crucial to consider the specific clinical scenario, including the degree of angulation, to determine the most suitable abutment type and framework material for each individual case. Understanding these factors is crucial for improving the longevity and success of dental implants. In accordance with various findings from prior research on the extent and pattern of fatigue performance on implants and bone across varying abutment angulations and prosthesis materials, further investigations are warranted in this area. Despite advances in dental implant technology, while the existing literature offers valuable insights into the individual effects of abutment type, material framework, and angulations on implant biomechanics, there is a clear gap in the research of comprehensively examining the combined impact of these factors. Therefore, the primary objective of this study was to evaluate the impact of abutment angulation, type, and diverse materials utilized for implant-supported prostheses on the stress distribution and fatigue performance within the implant and adjacent tissues through FEA. This study’s null hypothesis posited that abutment angulation and the use of different framework materials for implant-supported prostheses would not influence the fatigue performance.

## 2. Materials and Methods

### 2.1. Preparation of Specimens

**Figure 1:**
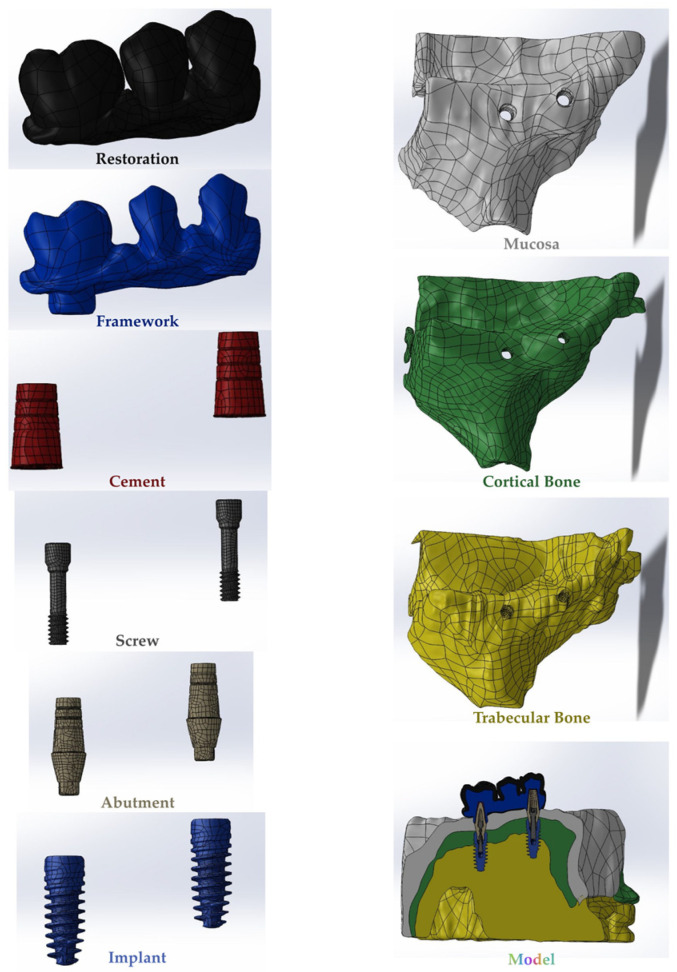
The final version of the obtained models.

**Figure 2:**
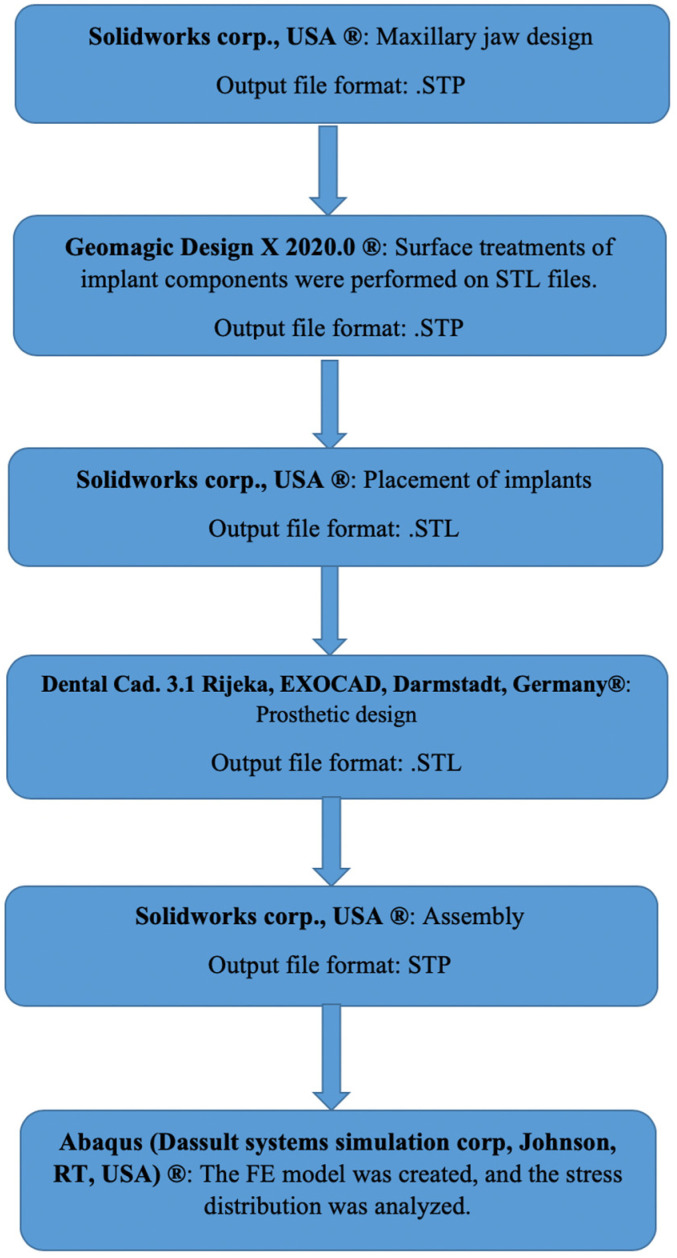
The key steps of the methodology.

In this study, multi-unit and resin-cemented restoration designs of Bil implant company (İstanbul, Türkiye) were used. Restorations with two different framework materials (porcelain fused to base metal (PFBM) and porcelain fused to base zirconia (PFBZ)) were used. Straight (3LSRAGH2NP, new ⌀3.7 mm implant L 10 mm), 15° (Abutment screw NP, BLSR156H2ANP, new ⌀3.7 mm implant L 10 mm), and 30° (Abutment screw NP, BLSR306H2ANP, new ⌀3.7 mm implant L 10 mm) abutments of the company were used for the multi-unit design. Additionally, for the resin-cemented type, straight (Abutment screw NP, new ⌀3.7 mm implant L 10 mm, PAD35GH2AH55NP), 15° (Abutment screw NP, new ⌀3.7 mm implant L 10 mm, PAA156H2AH7NP), and 25° (Abutment screw NP, new ⌀3.7 mm implant L 10 mm, PAA25GH2AH7NP) abutments were used. The groups used in the analysis are as follows: multi-unit 0-degree metal framework (Mu0M); multi-unit 15-degree metal framework (mu15M); multi-unit 30-degree metal framework (Mu30M); cemented 0-degree metal framework (C0M); cemented 15-degree metal framework (C15M); cemented 25-degree metal framework (C25M); multi-unit 0-degree zirconia framework (Mu0Z); multi-unit 15-degree zirconia framework (Mu15Z); multi-unit 30-degree zirconia framework (Mu30Z); cemented 0-degree zirconia framework (C0Z); cemented 15-degree zirconia framework (C15Z); and cemented 25-degree zirconia framework (C25Z). For the preparation of the specimens, a model was designed to mimic the maxillary jaw bone (mucosa, trabecular, and cortical bone) of an edentulous patient. The assembly module of the Solidworks 2013 (Solidworks Corp., Waltham, MA, USA) program was used for this design. In the design, the region of tooth 15 was considered missing and the bridge planning was aimed to be realized with implant placement in the region of teeth 14 and 16. Into the STP file that was obtained, the implants were positioned according to the information received from Bil implant company. In the insert section, the indent command in the feature section was used to create the cavity of the implant in the bone, and STL files were obtained for the prosthesis design. Following that, the designs of the prostheses were made using the Exocad program (Dental Cad. 3.1 Rijeka, EXOCAD, Darmstadt, Germany). These STL files were transferred to the Geomagic Design X program (Geomagic Design X 2020.0) and STP files were obtained after adjustments such as smoothing. The prostheses were then placed on the implants on the model. In addition, for resin-cemented restorations, a cement gap of 40 μm was closed with resin cement. The final version of the obtained models was saved as an STP file and transferred to the Abaqus program (ABAQUS 2020 Dassault Systems Simulation Corp., Johnston, RT, USA) for FEA (Figure 1). In the Abaqus program, the properties of the materials were first transferred to the program in the property section, and the analysis type was defined as static, general, and direct cyclic in the step section. In the direct cyclic section, the number of cycles was entered as 1000. Then, in the interaction section, the contacts (except for the contact between the screw and the implant) were transferred to the program as a tie and the loading and boundary conditions were performed in the load section. Then, the mesh process was performed in the mesh section of the Abaqus program (Figure 2). The total number of nodes and elements is shown in Table 1. Material properties were defined for all components to be included in the study (Table 2). The chosen properties reflect the typical mechanical behavior of every material as documented in previous studies, as also mentioned in Table 2. The connections between the components were provided in the interaction area. The connections simulated as a tie to simulate the osseointegration property were as follows: framework and porcelain, composite and prosthesis, bone and mucosa, and implant and bone. The connection between the screw parts was simulated by giving torque properties. 

### 2.2. Estimating the Stress Distribution 

A load of 150 N was applied to the occlusal table (an inclination from palatal to buccal, at an angle of 30 degrees). At this stage, the maxillary bone was encastred, which means that the maxillary bone was restricted from movement and rotation in all directions. In this study, it was assumed that the implants were completely osseointegrated with the bone and that the implant components did not show any movement on the implant surface. In order to define the quality of integration, a frictional contact type was applied between the abutment, fixture, and abutment screw interface. The frictional coefficient was set to 0.3. 

**Figure 3:**
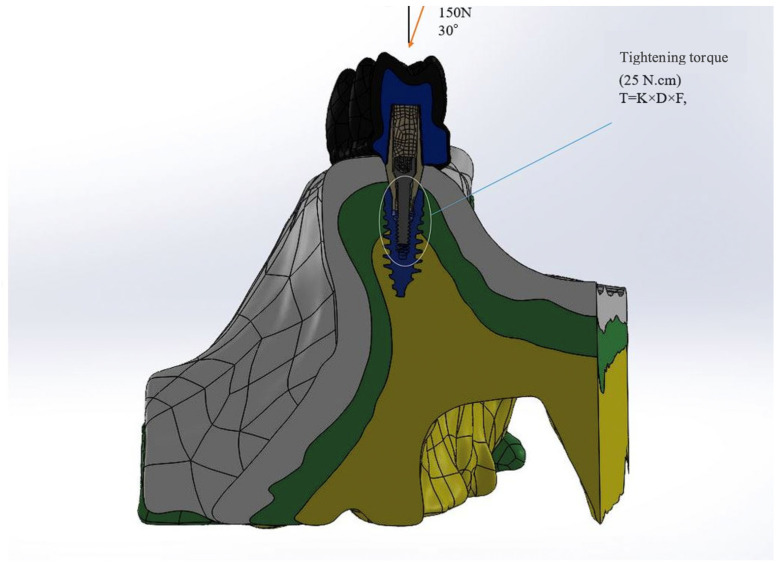
Screw preloading with the bone block fixed along the axes.

The equation for the screw torque moment is as follows:

(1) T=K×D×F,

where *T* corresponds to the screw torque moment (Ncm); *D* is the screw diameter (m); *F* is the screw preload (N); and *K* is the screw factor or torque coefficient (usually with a value of 0.2). Using the above equation, the screw preload value can be calculated if a tightening moment is applied to the abutment screw. In this study, using a tightening moment of 25 Ncm, the screw preload value was calculated as 781 N (Figure 3). After this process, stress values were obtained. Stress values are reported as von Mises Stress values (vMS).

### 2.3. Estimating the Fatigue Performance

For long-term implant success, maximum or endless fatigue performance needs to be achieved. As an evaluation method, physical testing or fatigue analysis can be applied. In this study, the following equations were used through FE modeling to estimate the fatigue performance of implants.

(2) $$\frac{\gamma }{2}+\frac{{∆\varepsilon }_{N}}{2}=1.65\frac{\sigma_{f}^{\prime }-\sigma_{N,m}}{E}{\left(2N_{f}\right)}^{b}+1.75\varepsilon_{f}^{\prime }{\left(2N_{f}\right)}^{c}.$$

where *σ_N,m_* corresponds to the normal stress on the critical plane; 2*N_f_* is the number of reversals to crack initiation; *γ*/2 is the shear strain amplitude; Δ*ε_N_* is the normal strain on the critical plane; $\sigma_{f}^{\prime }$ is the fatigue strength coefficient; $\varepsilon_{f}^{\prime }$ is the normal fatigue ductility coefficient; *E* is the elastic modulus; *c* is the fatigue ductility exponent; and *b* is the fatigue strength exponent. The fatigue ductility exponent and coefficient are derived from the Coffin–Manson law [51].

(3) $$\varepsilon_{P}=\varepsilon_{f}^{\prime }{\left(2N_{f}\right)}^{c}.$$

where $\varepsilon_{P}\;A$ represents the plastic strain. 

The fatigue strength exponent and coefficient come from Basquin’s law:

(4) $$\varepsilon_{e}=\frac{\sigma_{a}}{E}=\frac{\sigma_{f}^{\prime }}{E}{\left(2N_{f}\right)}^{b}.$$

(5) $$\sigma_{a}=\frac{∆\sigma }{2}=\frac{\left(\sigma_{max}-\sigma_{min}\right)}{2}.$$

where $\varepsilon_{e}$ is equivalent to the elastic component of the cyclic strain amplitude; ve $\sigma_{a}$ is equivalent to the cyclic stress amplitude.

The material properties were approximated using Seeger’s method by rescaling the conventional monotonic ultimate tensile stress (UTS) [52]. 

Table 3 shows the values of all the relative parameters for titanium according to Seeger’s method.

## 3. Results

### 3.1. Stress Distribution

#### 3.1.1. Stress Values on the Implant

As a result of this study, the lowest vMS values were generally seen in multi-unit restorations for implants in the premolar region. The lowest vMS was found in Mu0M. In resin-cemented restorations, where the vMS was higher than in multi-unit restorations, the highest vMS was seen in C25Z. For both types of abutments (multi-unit and resin cemented), vMS values increase with increasing angulation. In addition, PFBZ obtained higher vMS values for both restorations.

In the implants in the molar region, the lowest vMS was observed in Mu0M. The highest vMS was obtained in Mu30Z. In both types of restorations (multi-unit and resin cemented), regardless of whether PFBM or PFBZ was used, vMS increased with increasing angulation. For implants in the molar region, lower vMS values are observed up to 15 degrees of angulation in screw-retained restorations, regardless of the framework material. When the angulation increases to 30 degrees in multi-unit restorations, higher vMS values are observed compared to resin-cemented restorations with an angulation of 25 degrees. Similar to the premolar region, PFBZ has higher vMS for both restorations.

**Figure 4:**
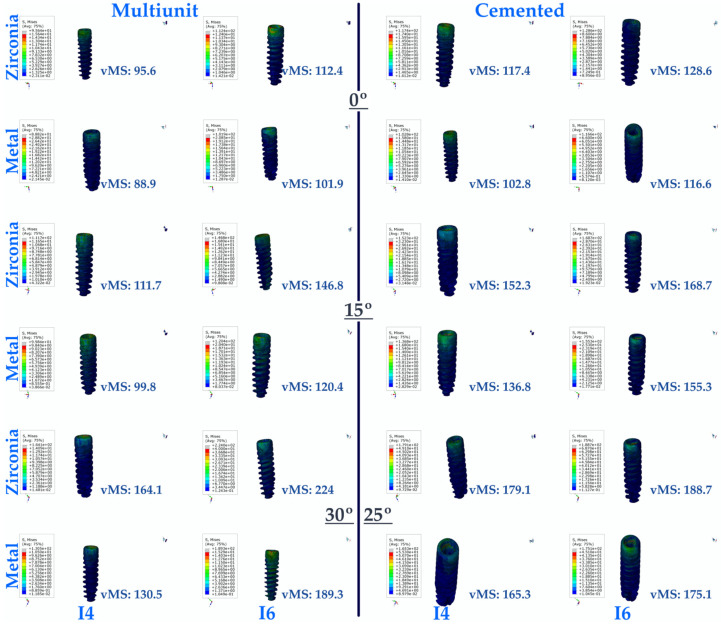
Von Mises Stress on the implant. vMS: von Mises Stress; I4: implant in the premolar area; I6: implant in the molar area.

In general, it was found that the vMS applied to implants in the premolar region was less distributed compared to implants in the molar region (Figure 4).

#### 3.1.2. Stress Values on the Abutment

The lowest vMS seen in the abutments in the premolar region was seen in Mu0M, while the highest vMS was seen in C25Z. When both resin-cemented and multi-unit restorations were evaluated separately, it was observed that the vMS on the abutments increased with increasing angulation. In addition, the highest vMS values were obtained in restorations with the highest angulation. When the vMS on the abutments in the premolar region was analyzed, a higher vMS was observed in restorations with PFBZ in direct proportion to the increase in angulation, while this distribution was lower in restorations with PFBM. It was observed that the vMS on the abutments in the premolar region was lower when multi-unit restorations were used compared to resin-cemented restorations. 

Similar to the abutments in the premolar region, the lowest vMS in the abutments in the molar region was observed in Mu0M, while the highest vMS was found in C25Z. In addition, the highest vMS values were obtained in restorations with the highest angulation. Furthermore, lower vMS values were obtained in multi-unit restorations compared to resin-cemented restorations regardless of the framework material. Again, PFBZ gave higher vMS than PFBM in every group. 

**Figure 5:**
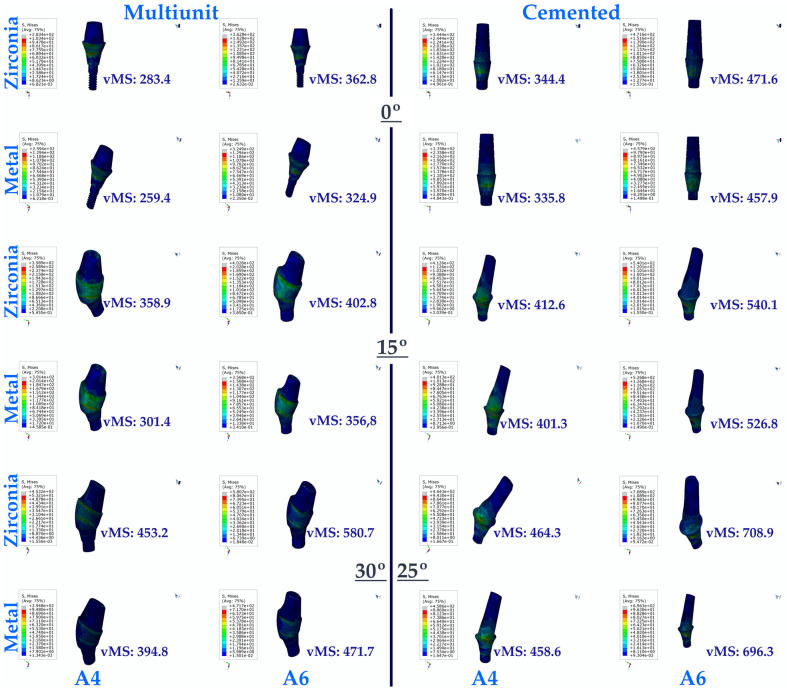
Von Mises Stress on the abutment. vMS: von Mises Stress; A4: abutment in the premolar area; A6: abutment in the molar area.

Among the vMS observed in the abutments, the lowest vMS values were observed in the premolar region, similar to the vMS observed in the implants (Figure 5).

#### 3.1.3. Stress Values on the Cortical Bone

**Figure 6:**
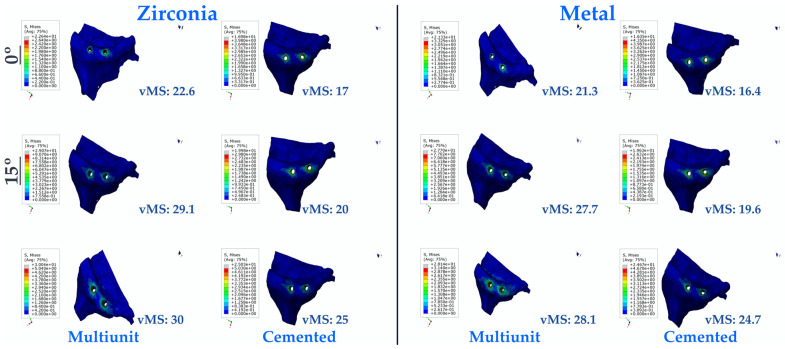
Von Mises Stress on the cortical bone. vMS: von Mises Stress.

As a result of this study, the effects of the applied stress on the cortical bone were also examined. The lowest vMS was observed in C0M, while the highest vMS was observed in Mu30Z. In both multi-unit and resin-cemented restorations, regardless of the framework material, it was found that vMS increased with increasing angulation. In addition, vMS values in bone were lower in resin-cemented restorations, while higher vMS values were obtained in multi-unit restorations. In addition, all PFBZ restorations calculated higher vMS values than PFBM restorations (Figure 6).

#### 3.1.4. Stress Values on the Framework

**Figure 7:**
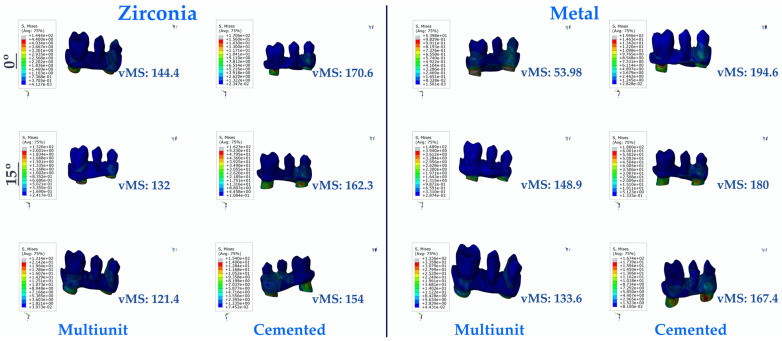
Von Mises Stress on the framework. vMS: von Mises Stress.

When the prosthetic frameworks were analyzed, the lowest vMS was found in Mu30Z. The highest vMS was observed in C0M. In both types of restorations, vMS decreased with increasing angulation, independent of the framework material. Multi-unit restorations were found to have lower vMS than resin-cemented restorations. In addition, in both types of restorations, PFBM and PFBZ restorations caused a decrease in vMS inversely proportional to the increase in angulation, while PFBM restorations gave higher vMS values than PFBZ restorations (Figure 7).

#### 3.1.5. Stress Values on the Abutment Screw

When the vMS on the abutment/multi-unit screw were analyzed, the highest vMS was found in Mu30Z. The lowest vMS was also seen in multi-unit restorations. Among these restorations, PFBM restorations with a 15-degree angle were found to have the lowest vMS. In direct proportion to the increase in angulation, there is an increase in the vMS values of the abutment/multi-unit screw in both PFBZ and PFBM restorations. The vMS values of the abutment/multi-unit screw of PFBM restorations were lower than PFBZ restorations in all angle groups. In the 15-degree-angle groups, screw-retained restorations showed lower vMS, regardless of the framework. With increasing angulation, multi-unit restorations were found to have higher vMS than resin-cemented restorations. 

When the vMS on the abutment/multi-unit screw in the molar region was examined, the highest vMS was seen for C25Z. In both regions (premolar and molar), the lowest vMS was found in C15M. In direct proportion to the increase in angulation, an increase in the vMS to the abutment/multi-unit screw was observed in both PFBZ and PFBM restorations. The vMS to the abutment/multi-unit screw of PFBM restorations were lower than those of PFBZ restorations in all angle groups. Multi-unit restorations showed lower vMS regardless of the framework material, while resin-cemented restorations obtained higher vMS values in each group.

The resultant vMS of the abutment/multi-unit screw in the restoration to the applied stress was lower in each angulation, restoration type, and framework material group in the premolar region.

#### 3.1.6. Stress Values on the Occlusal Screw

The highest vMS on the occlusal screws of multi-unit restorations in the premolar region was observed in the occlusal screw of Mu30Z, while the lowest vMS was observed in the occlusal screw of Mu0M. In multi-unit restorations, an increase in occlusal screws in both frameworks was observed in direct proportion to the increase in angle. The vMS values of the occlusal screws in PFBM restorations were lower than the occlusal screw vMS values in PFBZ restorations.

In the molar area, results matching the premolar area were obtained. The lowest occlusal screw vMS was seen in Mu0M, while the highest vMS was found in Mu30Z. Similarly, in this region, the resultant vMS on the occlusal screw increased with increasing angles. The occlusal screw vMS values of PFBZ restorations were higher than those of PFBM restorations. 

**Figure 8:**
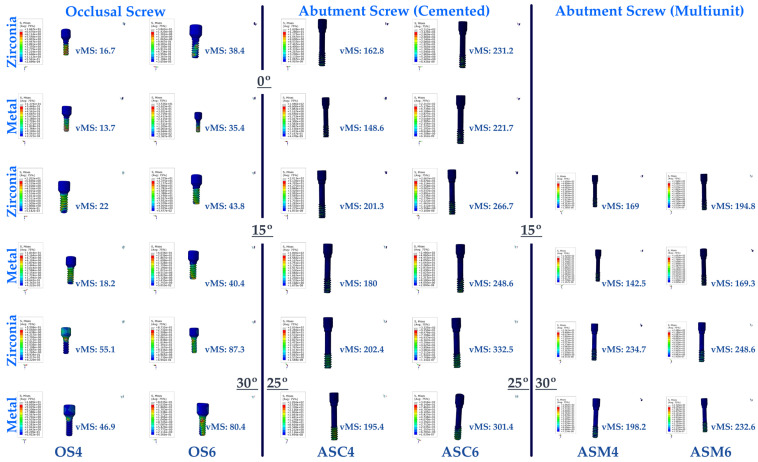
Von Mises Stress on the screw. vMS: von Mises Stress; OS4: occlusal screw in the premolar area; OS6: occlusal screw in the molar area; ASC4: abutment screw of cemented restoration in the premolar area; ASC6: abutment screw of cemented restoration in the molar area; ASM4: abutment screw of multi-unit restoration in the premolar area; ASM6: abutment screw of multi-unit restoration in the molar area.

The result of the applied stress on the occlusal screws was found to be lower in the premolar region than in the molar region in all framework and angulation groups (Figure 8).

#### 3.1.7. Stress Values on the Resin Cement

When the vMS on the cement in resin-cemented restorations were analyzed, the highest vMS in the premolar region was observed in C0M. In addition, the lowest vMS was found in C25Z. An inversely proportional decrease in the vMS on the resin cement was observed in both PFBM and PFBZ restorations with increasing angles. The vMS values in the resin cements of PFBM restorations were higher than those of PFBZ restorations in each angle group.

In the resin cement vMS of restorations in the molar region, the lowest data were obtained in C0Z. The highest vMS was found in C25M. Both frameworks of restorations show an increase in resin cement vMS with increasing angles. It was determined that the resin cement vMS values of PFBM restorations were higher than PFBZ restorations.

**Figure 9:**
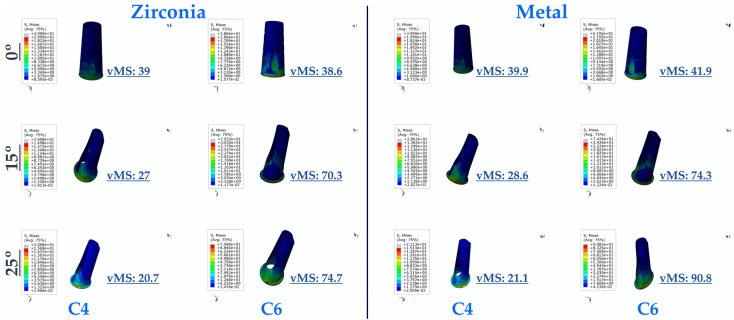
Von Mises Stress on the resin cement. vMS: von Mises Stress; C4: resin cement in the premolar area; C6: resin cement in the molar area.

The resultant vMS of the resin cement in the restoration to the applied stress was generally lower in the premolar groups. Differently, in C0Z, slightly higher resin cement vMS values were obtained in the premolar region than in the molar region (Figure 9).

### 3.2. Fatigue Performance 

#### 3.2.1. Fatigue Performance of Implant

**Figure 10:**
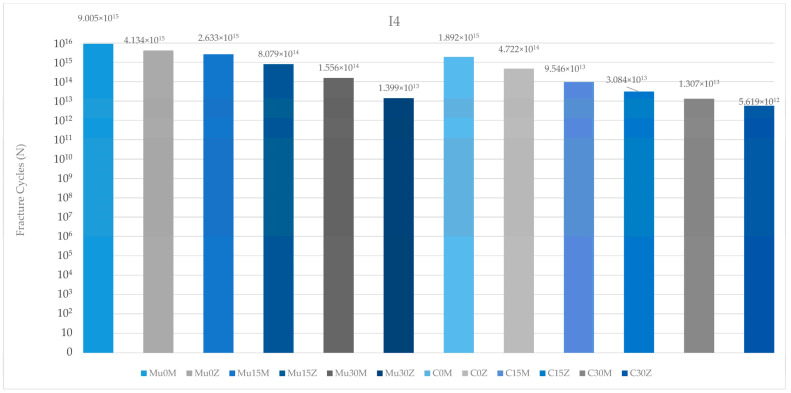
Fracture cycles of implants in the premolar region. I4: implant in the premolar area; Mu0M: multi-unit 0-degree metal framework; Mu0Z: multi-unit 0-degree zirconia framework; Mu15M: multi-unit 15-degree metal framework; Mu15Z: multi-unit 15-degree zirconia framework; Mu30M: multi-unit 30-degree metal framework; Mu30Z: multi-unit 30-degree zirconia framework; C0M: cemented 0-degree metal framework; C0Z: cemented 0-degree zirconia framework; C15M: cemented 15-degree metal framework; C15Z: cemented 15-degree zirconia framework; C25M: cemented 25-degree metal framework; C25Z: cemented 25-degree zirconia framework.

According to the data obtained as a result of this study, the highest implant fatigue performance in the premolar region was observed in Mu0M. On the other hand, the poorest implant fatigue performance was recorded in C25Z. Regardless of the type of abutment (multi-unit or resin cemented), fatigue performance decreased inversely with increasing angles. Again, in both restoration types, implants with PFBZ showed lower values than implants with PFBM. Multi-unit restorations were more successful than resin-cemented restorations (Figure 10).

**Figure 11:**
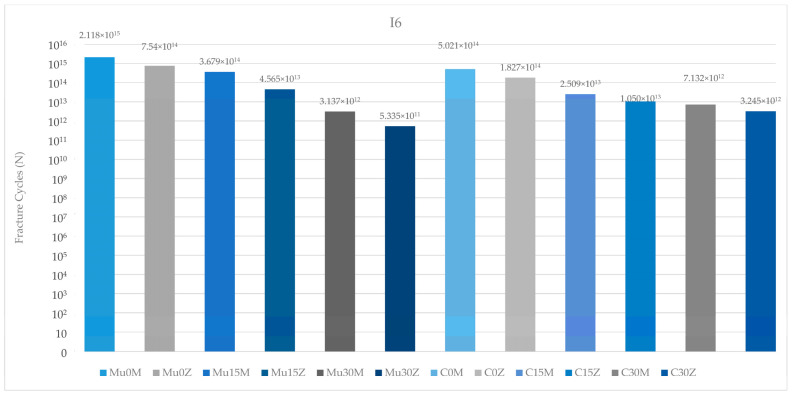
Fracture cycles of implants in the molar region. I6: implant in the molar area; Mu0M: multi-unit 0-degree metal framework; Mu0Z: multi-unit 0-degree zirconia framework; Mu15M: multi-unit 15-degree metal framework; Mu15Z: multi-unit 15-degree zirconia framework; Mu30M: multi-unit 30-degree metal framework; Mu30Z: multi-unit 30-degree zirconia framework; C0M: cemented 0-degree metal framework; C0Z: cemented 0-degree zirconia framework; C15M: cemented 15-degree metal framework; C15Z: cemented 15-degree zirconia framework; C25M: cemented 25-degree metal framework; C25Z: cemented 25-degree zirconia framework.

Regarding the fatigue performance of the implants in the molar region, the highest value was seen in the metal-framework restoration with no angle, while the lowest value was found in Mu30Z. As in the premolar region, an increase in the angle—independent of the restoration and framework material—led to a decrease in fatigue performance. In both frameworks, the implant in the multi-unit restoration generally achieved higher values than the implant in the resin-cemented restoration. In addition, the implant in C25M performed better than the implant in Mu30M. PFBZ restorations showed lower fatigue performance than the implants of PFBM restorations in each group (Figure 11).

Considering the fatigue performance of the implants in both regions, the results in the premolar region were more successful than the results in the molar region. 

#### 3.2.2. Fatigue Performance of Abutment

**Figure 12:**
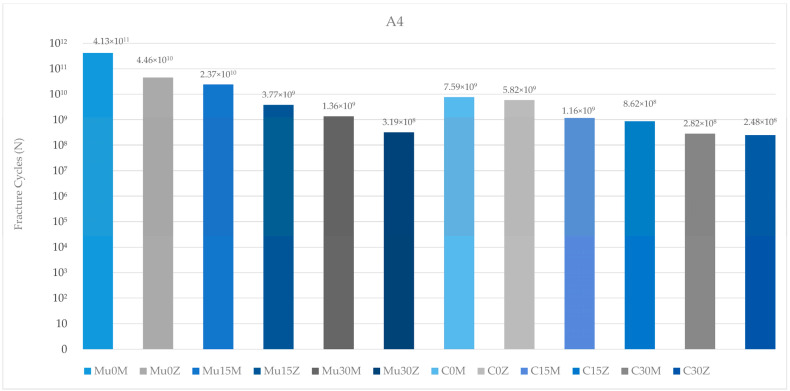
Fracture cycles of abutments in the premolar region. A4: abutment in the premolar area; Mu0M: multi-unit 0-degree metal framework; Mu0Z: multi-unit 0-degree zirconia framework; Mu15M: multi-unit 15-degree metal framework; Mu15Z: multi-unit 15-degree zirconia framework; Mu30M: multi-unit 30-degree metal framework; Mu30Z: multi-unit 30-degree zirconia framework; C0M: cemented 0-degree metal framework; C0Z: cemented 0-degree zirconia framework; C15M: cemented 15-degree metal framework; C15Z: cemented 15-degree zirconia framework; C25M: cemented 25-degree metal framework; C25Z: cemented 25-degree zirconia framework.

When the fatigue performance of the abutments in the premolar region was analyzed, the highest value was found in Mu0M, while the lowest value was found in C25Z. In both restoration types (multi-unit and resin cemented), the fatigue performance of the abutments in the PFBM group was higher than the abutments in the PFBZ group. Increasing the angles caused a decrease in the fatigue performance of the abutments in each group. The abutments in the multi-unit restoration achieved higher performance results in each framework group than the abutments in the resin-cemented restoration (Figure 12).

**Figure 13:**
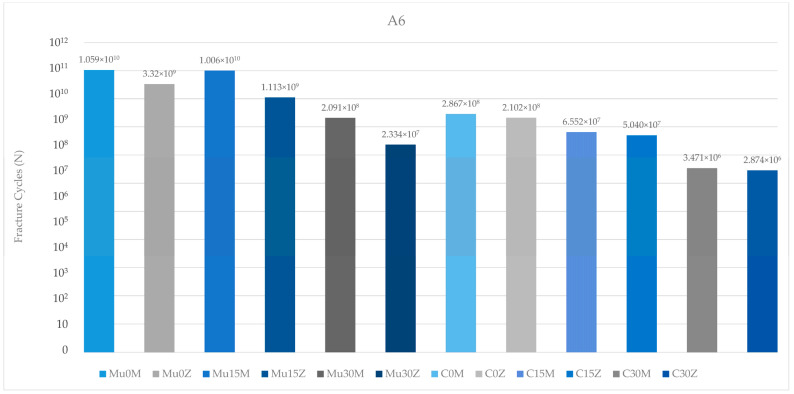
Fracture cycles of abutments in the molar region. A6: abutment in the molar area; Mu0M: multi-unit 0-degree metal framework; Mu0Z: multi-unit 0-degree zirconia framework; Mu15M: multi-unit 15-degree metal framework; Mu15Z: multi-unit 15-degree zirconia framework; Mu30M: multi-unit 30-degree metal framework; Mu30Z: multi-unit 30-degree zirconia framework; C0M: cemented 0-degree metal framework; C0Z: cemented 0-degree zirconia framework; C15M: cemented 15-degree metal framework; C15Z: cemented 15-degree zirconia framework; C25M: cemented 25-degree metal framework; C25Z: cemented 25-degree zirconia framework.

The results of the abutments in the molar region generally match the results of the abutments in the premolar region. Again, the highest value was seen in Mu0M, while the lowest value was found in C25Z. In both types of abutments (multi-unit and resin cemented), there was a decrease in values inversely proportional to the increase in angle. Contrary to this result, the abutment in Mu15M showed higher fatigue performance than the abutment in Mu0Z. Similar to the abutments in the premolar region, the abutments in this region also achieved better fatigue performance in both frameworks in multi-unit restorations. On the other hand, the fatigue performance of abutments in PFBZ restorations was lower in each group (Figure 13).

When the fatigue performance of the abutments was analyzed, the results in the premolar region were found to be higher than in the molar region.

#### 3.2.3. Fatigue Performance of Abutment Screw

**Figure 14:**
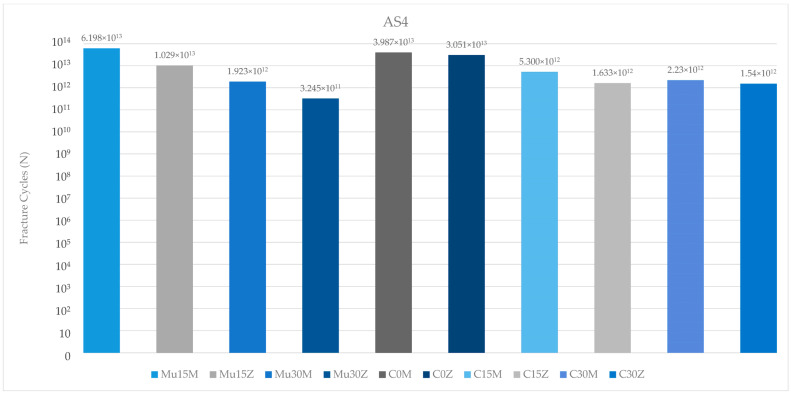
Fracture cycles of abutment screws in the premolar region. AS4: abutment screw in the premolar area; Mu0M: multi-unit 0-degree metal framework; Mu0Z: multi-unit 0-degree zirconia framework; Mu15M: multi-unit 15-degree metal framework; Mu15Z: multi-unit 15-degree zirconia framework; Mu30M: multi-unit 30-degree metal framework; Mu30Z: multi-unit 30-degree zirconia framework; C0M: cemented 0-degree metal framework; C0Z: cemented 0-degree zirconia framework; C15M: cemented 15-degree metal framework; C15Z: cemented 15-degree zirconia framework; C25M: cemented 25-degree metal framework; C25Z: cemented 25-degree zirconia framework.

Regarding the fatigue performance of the abutment screws used in this study in the premolar region, the highest value was seen in Mu15M. The lowest value was found in the abutment screw of Mu30Z. Increasing the angle caused a decrease in the fatigue performance of the abutment screws. In addition, compared to the abutment screws of multi-unit and resin-cemented restorations with the same angle (15 degrees), multi-unit restorations were found to be higher in both frameworks. In both types of abutments (multi-unit and resin cemented), abutment screws in PFBZ were found to have lower fatigue performance (Figure 14). 

**Figure 15:**
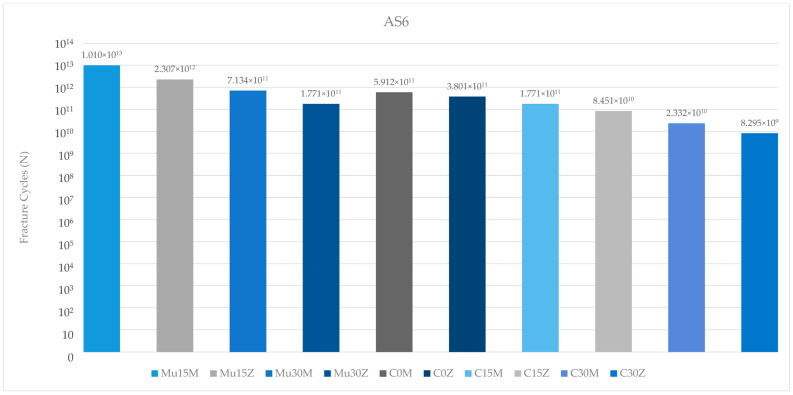
Fracture cycles of abutment screws in the molar region. AS6: abutment screw in the molar area; Mu0M: multi-unit 0-degree metal framework; Mu0Z: multi-unit 0-degree zirconia framework; Mu15M: multi-unit 15-degree metal framework; Mu15Z: multi-unit 15-degree zirconia framework; Mu30M: multi-unit 30-degree metal framework; Mu30Z: multi-unit 30-degree zirconia framework; C0M: cemented 0-degree metal framework; C0Z: cemented 0-degree zirconia framework; C15M: cemented 15-degree metal framework; C15Z: cemented 15-degree zirconia framework; C25M: cemented 25-degree metal framework; C25Z: cemented 25-degree zirconia framework.

Among the fatigue performance of the abutment screws in the molar region, the highest value was found in Mu15M. The lowest abutment screw fatigue performance was found in C25Z. In all groupings, fatigue performance of the abutment screw decreased inversely with increasing angles. In multi-unit restorations, abutment screws exhibited higher fatigue performance than the resin-cemented groups. In addition, the abutment screws of PFBZ restorations always showed lower values than the PFBM groups (Figure 15). 

Regarding the fatigue performance of the abutment screws, higher values were found in the premolar region than in the molar region, independently of the group.

#### 3.2.4. Fatigue Performance of Occlusal Screw

**Figure 16:**
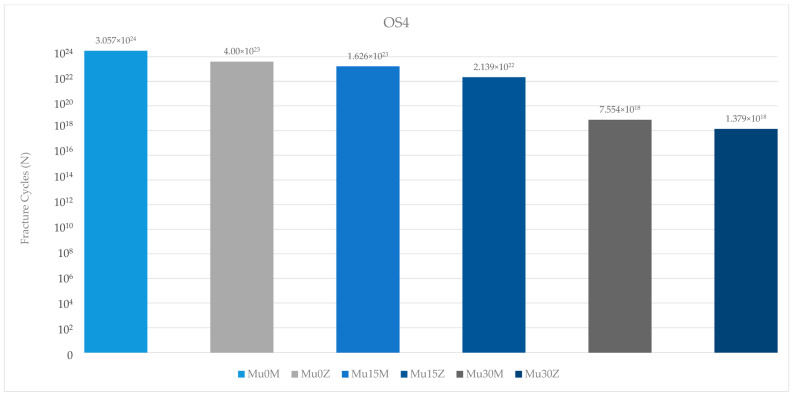
Fracture cycles of occlusal screws in the premolar region. OS4: occlusal screw in the premolar area; Mu0M: multi-unit 0-degree metal framework; Mu0Z: multi-unit 0-degree zirconia framework; Mu15M: multi-unit 15-degree metal framework; Mu15Z: multi-unit 15-degree zirconia framework; Mu30M: multi-unit 30-degree metal framework; Mu30Z: multi-unit 30-degree zirconia framework; C0M: cemented 0-degree metal framework; C0Z: cemented 0-degree zirconia framework; C15M: cemented 15-degree metal framework; C15Z: cemented 15-degree zirconia framework; C25M: cemented 25-degree metal framework; C25Z: cemented 25-degree zirconia framework.

When the fatigue performance values of the occlusal screws in the premolar region were analyzed, the highest value was found in Mu0M. The lowest value was found in Mu30Z. An inversely proportional decrease in occlusal screw fatigue performance was observed with increasing angles, regardless of the framework. The occlusal screw in the PFBZ group showed a lower fatigue value than the PFBM restoration (Figure 16). 

**Figure 17:**
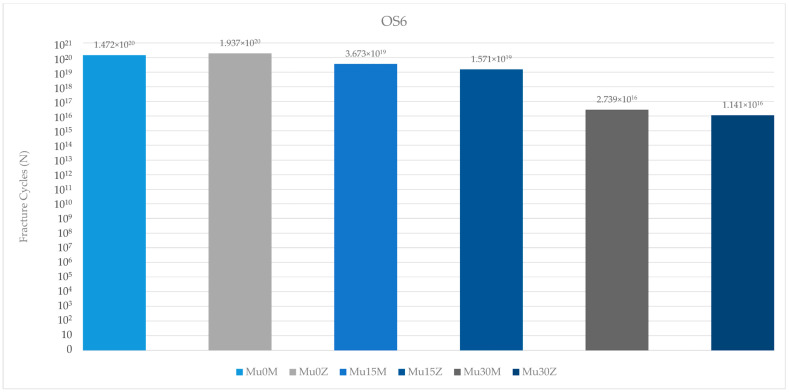
Fracture cycles of occlusal screws in the molar region. OS6: occlusal screw in the molar area; Mu0M: multi-unit 0-degree metal framework; Mu0Z: multi-unit 0-degree zirconia framework; Mu15M: multi-unit 15-degree metal framework; Mu15Z: multi-unit 15-degree zirconia framework; Mu30M: multi-unit 30-degree metal framework; Mu30Z: multi-unit 30-degree zirconia framework; C0M: cemented 0-degree metal framework; C0Z: cemented 0-degree zirconia framework; C15M: cemented 15-degree metal framework; C15Z: cemented 15-degree zirconia framework; C25M: cemented 25-degree metal framework; C25Z: cemented 25-degree zirconia framework.

When the occlusal screw fatigue performance in the molar region was examined, the most successful value was found in Mu0Z. The poorest fatigue value was found in the occlusal screw of Mu30Z. In the occlusal screw of both types of framework restorations; increasing the angle caused a decrease in fatigue performance. Only in the group without an angle, the performance of the occlusal screw in the PFBZ obtained a higher value (Figure 17). 

When the occlusal screw fatigue performance in both regions was analyzed, the values in the premolar region were more successful in both framework groups.

## 4. Discussion

This study evaluated the effect of the angle and type of abutments and the composition of the restoration framework on the stress distribution and fatigue performance of dental implant components. The null hypothesis was rejected. The composition of the crown framework material appears to influence the stress distribution and fatigue performance of the abutment–implant complex. In addition, high implant angulation also influenced stress distribution and fatigue performance. 

The annual chewing cycle of an individual is recognized as 1,130,000 [55,56]. The results of the study were evaluated accordingly. Studies investigating the fatigue performance of dental restorations in molar and premolar regions are essential for understanding the durability and longevity of these treatments. Lovera-Prado et al. [57], found that dental implant fatigue limits differ across tooth regions, with premolars falling between incisors and molars in terms of values. Other studies have shown that the occlusal forces in the molar region can range from 250 to 400 N, while in the premolar region, they can vary from 140 to 170 N [58]. Consistent with the findings of these studies, in this study, lower fatigue performance values were obtained for implant components in the molar region than in the premolar region. As a result, it can be said that this part of this study is in line with the literature. The fatigue performance of abutments is also a critical aspect in implant dentistry. Consistent with studies reporting that fatigue failure will occur at the weakest point of the implant, the earliest deformation in the study was seen on the abutment in the molar region [59,60]. In addition, the highest fatigue strength was observed in the occlusal screw in the premolar region. The fatigue resistance of occlusal screws is influenced by various factors such as design, material, and loading conditions [61]. Additionally, the influence of the vertical misfit on screw fatigue behavior has been investigated, highlighting the importance of proper fit to prevent fatigue failure [62]. Proper insertion and the torquing of screws have been studied to assess their effect on screw loosening and fatigue resistance [63]. Additionally, the diameter of the occlusal access hole in screw-retained prostheses can affect the distribution of occlusal forces and potentially impact fatigue performance [64]. In conclusion, although it is unlikely to be feasible in real life due to physician or material manufacturing errors, as demonstrated in this study, the flawless application of occlusal screws is important for longevity and durability. Again, it is seen that these results are similar to the literature. Moreover, as expected, a decrease in fatigue strength was observed in each component as the angle increased. The lower stress observed at lower angles (0–15 degrees) suggests that there is a potential to reduce implant failure rates in clinical settings, highlighting the need for more precise angle assessments during surgical procedures. Multiple studies indicate a correlation between angle and fatigue properties [65,66,67,68]. Also, another study that underlines the importance of geometric parameters, such as angles, in influencing the fatigue properties of mechanical components, supports the results of the study [69]. This study suggests that the correct choice of framework material and restoration type will become more important as the angle increases. Multi-unit restorations have been a topic of interest in dental research, particularly in comparison to resin cement-retained restorations. Several studies have investigated the success rates and complications associated with these two types of restorations [70,71]. To determine the comparative fatigue performance of multi-unit versus resin-cemented restorations, it is crucial to consider studies that specifically address the fatigue behavior of these restorative techniques. In general, in this study, superior fatigue performance was obtained in the use of multi-unit restorations compared to resin-cemented restorations. Resin-cemented restorations obtained higher values only of the implant in the molar region and the abutment screw in the premolar region. The incompatibility of the angles between the two restoration types is thought to be the reason for this. As the angle increases, the choice of restoration type requires precision. Multi-unit restorations show superior success compared to resin-cemented restorations, even in cases with high angulations. This conclusion is also supported by the superior fatigue performance of the occlusal screw in the premolar region. As a result, it can be said that this part of this study is also in line with the literature. Based on this information, it can be concluded that multi-unit restorations should be preferred, especially in cases with large angulations. This could reduce stress levels in implants placed at increased angles. However, it should be noted that in this study, multi-unit restorations achieved superior fatigue performance compared to resin-cemented restorations, regardless of the angle. Moreover, the type of restoration framework can affect fatigue behavior. When PFBZ and PFBM materials were evaluated in this study, in general, PFBM restorations showed higher fatigue performance. Similar to this study, Jung et al. [72] discovered that the survival rate of metal–ceramic crowns was notably higher than that of all-ceramic crowns, indicating better longevity for metal–ceramic restorations. Similarly, Sailer et al. [73] observed a lower complication rate for PFBM fixed dental prostheses compared to PFBZ prostheses, further supporting the superior performance of metal–ceramic restorations in terms of durability. Furthermore, Hosseini et al. [74] pointed out a higher incidence of veneering fractures in zirconia crowns compared to metal–ceramic crowns, suggesting that metal–ceramic restorations may be more resistant to fatigue-related failures. This is consistent with the findings of Bajunaid et al. [75], who noted that ceramic materials, including zirconia, are susceptible to cohesive fractures and chipping, indicating potential weaknesses in zirconia restorations under fatigue conditions. As a result, it can be said that this part of this study is in line with the literature, too. Overall, the synthesis of these studies suggests that implant-supported metal–ceramic restorations may indeed offer better fatigue performance than implant-supported zirconia restorations. The evidence points toward the higher survival rates, lower complication rates, and a reduced incidence of veneering fractures associated with metal–ceramic restorations, highlighting their potential as a more durable option for long-term success in dental implant applications. Finite element analysis (FEA) was utilized in this study to explore the research hypothesis. FEA is a valuable computational tool commonly employed in the examination of various aspects related to dental restorations, biomaterials, restorative techniques, and prosthetic designs, particularly focusing on stress distribution under different loading conditions. This numerical simulation method enables a comprehensive investigation and quantification of the biomechanical responses demonstrated by complex dental structures. It is frequently utilized to analyze stress patterns in dental biomechanical studies. The modeling process assumed perfect joining of all components in different configurations and the material properties were considered isotropic and linear. Ignoring any potential errors that may occur during material production and preparation, the calculation of just 1000 cycles and use of finite element analysis may not fully capture the complex biomechanical interactions in vivo and are among the limitations of the present study. In future studies, it is recommended to verify the obtained findings with in vivo studies and to use higher cycle values to increase the reliability of the results.

## 5. Conclusions

This study aims to address an important research gap and provide a comprehensive perspective on the complex rehabilitation of implant-supported resin-cemented or screw-retained restorations where an angle is present, and to provide valuable information for clinical practice. Direct comparisons of fatigue performance between screw-retained and resin-cemented restorations are limited in the literature. This study eliminated this uncertainty. Thus, it contributes to the literature.

The results of this study are as follows:

- In general, and especially in the case of implant restorations with an increased angle, the choice of multi-unit restorations will greatly increase the survival rate;
- PFBZ does not offer an ideal treatment alternative for implant restorations. Implant restorations with PFBM are currently still a better alternative;
- Occlusal screws are the most durable components when each stage is performed perfectly, in accordance with the procedure;
- Considering the chewing force on the molar region, posterior implant planning should be performed precisely;
- The placement of implants with a straight angle should be considered among the basic steps for the success of prosthetic treatments and surgical procedures should be carried out with sensitivity in this regard.

## Figures and Tables

**Table 1:** Total number of nodes, elements, and mesh types of 3 cemented and 3 multi-unit models.

Model	Total Number of Nodes	Total Number of Elements	Mesh Types
C0	2,884,871	15,083,472	Linear tetrahedral elements of type C3D4
C15	2,378,953	12,324,461	Linear tetrahedral elements of type C3D4
C25	2,032,783	10,586,603	Linear tetrahedral elements of type C3D4
Mu0	1,964,012	10,388,715	Linear tetrahedral elements of type C3D4
Mu15	2,301,340	12,108,325	Linear tetrahedral elements of type C3D4
Mu30	2,516,898	13,482,386	Linear tetrahedral elements of type C3D4

**Table 2:** Material properties assigned to the model.

Material	Young’s Modulus (E MPa)	Poisson’s Ratio (ν)	Shear Modulus (G Mpa)	References
Cortical Bone	E_x_ 12,600 E_y_ 12,600 E_z_ 19,400	ν_xy_ 0.300 ν_yz_ 0.253 ν_xz_ 0.253 ν_yx_ 0.300 ν_zy_ 0.390 ν_zx_ 0.390	G_xy_ 4850 G_yz_ 5700 G_xz_ 5700	[46]
Trabecular Bone	E_x_ 1148 E_y_ 210 E_z_ 1148	ν_xy_ 0.055 ν_yz_ 0.010 ν_xz_ 0.322 ν_yx_ 0.010 ν_zy_ 0.055 ν_zx_ 0.322	G_xy_ 68 G_yz_ 68 G_xz_ 434	[46]
Mucosa	2.8	0.40		[47]
Titanium	110,000	0.33		[48]
Felspathic Porcelain	82,800	0.28		[48]
Zirconia	210,000	0.30		[49]
Co-Cr Alloy	218,000	0.33		[47]
Resin Composite	12,000	0.33		[50]
Resin Cement	5100	0.27		[50]

**Table 3:** Material parameters using Seeger’s method [53,54].

$\sigma_{f}^{\prime }$	$\varepsilon_{f}^{\prime }$	*b*	*c*
1554.71	0.35	−0.095	−0.69

## Data Availability

The raw data supporting the conclusions of this article will be made available by the authors upon request.
